# Prevention of Recurrent Spontaneous Preterm Delivery Using Probiotics (Clostridium butyricum, Enterococcus faecium, and Bacillus subtilis; PPP Trial): Protocol for a Prospective, Single-Arm, Nonblinded, Multicenter Trial

**DOI:** 10.2196/59928

**Published:** 2024-09-09

**Authors:** Satoshi Yoneda, Tohru Kobayashi, Kayoko Kikuchi, Shintaro Iwamoto, Tsuyoshi Teramoto, Daisuke Chujo, Katsufumi Otsuki, Akihito Nakai, Shigeru Saito

**Affiliations:** 1 Department of Obstetrics and Gynecology University of Toyama Toyama Japan; 2 Department of Data Science Clinical Research Center National Center for Child Health and Development Tokyo Japan; 3 Center for Translational Research, Translational Research Headquarters Fujita Health University Aichi Japan; 4 Biostatistics Unit, Department of Data Science Clinical Research Center National Center for Child Health and Development Tokyo Japan; 5 Center for Clinical Research Toyama University Hospital Toyama Japan; 6 Department of Obstetrics and Gynecology Showa University Koto Toyosu Hospital Tokyo Japan; 7 Department of Obstetrics and Gynecology Nippon Medical School Tama-Nagayama Hospital Tokyo Japan; 8 University of Toyama Toyama Japan

**Keywords:** clostridium, multicenter open label trial, preterm delivery, probiotics, recurrent spontaneous preterm delivery, prospective single-arm, spontaneous preterm delivery, infection, oral probiotics, pregnant women, pregnant, pregnancy, neonates, preterm births, systematic review, meta-analysis

## Abstract

**Background:**

The rate of recurrent spontaneous preterm delivery (sPTD) ranges between 27% and 34% and is 22.3% in Japan. Although it currently remains unclear whether probiotics prevent sPTD, retrospective studies recently reported a reduction in the rate of recurrent sPTD with the administration of probiotics including *Clostridium* spp., which induce regulatory T cells that play an important role in maintaining pregnancy.

**Objective:**

The objective of this trial is to evaluate the preventative effects of available oral probiotics, including *Clostridium butyricum*, on recurrent sPTD.

**Methods:**

This is a prospective, single-arm, nonblinded, multicenter trial in Japan. The sample size required for this trial is 345 pregnant women with a history of sPTD, considering a clinically significant reduction in the relative risk of 30% (risk ratio=0.7). The primary endpoint is the rate of recurrent sPTD at <37 weeks of gestation. The secondary endpoints are the rate of sPTD at <34 weeks of gestation, the rate of recurrent sPTD at <28 weeks of gestation, the ratio of intestinal *Clostridium* spp. (detected by next-generation sequencing), and bacterial vaginosis (using the Nugent score).

**Results:**

The trial procedures were approved by the Clinical Research Review Board of Toyama University Hospital (SCR2020008) on March 31, 2021. The trial was registered on the Japan Registry of Clinical Trial website on April 28, 2021. Recruitment began on May 1, 2021, and the trial is estimated to finish on March 31, 2025.

**Conclusions:**

The findings will clarify the rate of recurrent sPTD following probiotic administration including *Clostridium butyricum*. Outcomes from this trial will inform clinical practice and guide future randomized controlled trials.

**Trial Registration:**

Japan Registry of Clinical Trials jRCTs041210014; https://jrct.niph.go.jp/latest-detail/jRCTs041210014

**International Registered Report Identifier (IRRID):**

DERR1-10.2196/59928

## Introduction

According to a systemic analysis, 13.4 million (12.3-15.2 million) live neonates were estimated to be delivered as preterm births (<37 weeks) in 2020 (9.9% [9.1%-11.2%] of all births) worldwide [1]. In addition, the number of neonate (aged 0 days to 27 days) deaths due to preterm birth complications is estimated at 938,000 [2]. Preterm neonates are at an increased risk of mortality and morbidity, such as sepsis, periventricular leukomalacia (PVL), intraventricular hemorrhage (IVH), cerebral palsy, seizures, bronchopulmonary dysplasia (BPD), necrotizing enterocolitis (NEC), feeding difficulties, and visual or hearing disorders [3]. Between 30% and 35% of preterm births are indicated, 65% to 70% are spontaneous preterm deliveries (sPTD) [4], and the rate of recurrent sPTD ranges between 27% and 34% [5]. In Japan, the rate of recurrent sPTD was recently reported to be 22.3%, based on real-world data from 2014 to 2016 [6].

sPTD is caused by multiple pathological processes [4,5,7], with intra-amniotic inflammation or infection being the main cause [7-9]. As the clinical characteristics of sPTD, severe intra-amniotic inflammation and infection were more frequent in the earlier weeks of gestation [8-10]. Since preterm labor, the preterm premature rupture of membranes, and cervical insufficiency develop due to the collapse of the intrauterine environment, such as by inflammation or infection, it is difficult to avoid sPTD after the appearance of clinical symptoms. Therefore, precautions are needed to prevent sPTD in pregnant women at a high risk of sPTD starting in the first trimester or before pregnancy.

In 2013, alliums (garlic, onion, leek, and spring onion) and dried fruits (raisins, apricots, prunes, figs, and dates) were reported to be associated with a decreased risk of sPTD [11]. In another prospective cohort study reported in 2019, intake of fermented food, such as miso soup, yogurt, and soybeans, before pregnancy significantly reduced the risk of early sPTD at <34 weeks [12]. According to a large-scale, epidemiological, observational, cohort study conducted in Norway, the consumption of probiotic milk during early pregnancy was correlated with a lower risk of preterm delivery (adjusted odds ratio 0.79, 95% CI 0.64-0.97; *P*=.03) [13]. As one of the pathological characteristics in the intestinal flora of pregnant women with sPTD, the abundance of *Clostridium* spp. was significantly lower than in pregnant women who delivered at term [14]. These findings of large-scale epidemiological studies and the specificity of the intestinal flora strongly suggest the potential of prebiotics or probiotics to improve the intestinal microbiota and prevent sPTD.

On the other hand, clinical studies using probiotics have not been sufficiently powered to prevent sPTD [15]. The findings of a systematic review and meta-analysis showed that the consumption of probiotics or prebiotics during pregnancy did not affect the risk of sPTD [16,17]. However, the types of probiotics or prebiotics used in each trial varied. Moreover, the initiation of their administration was not constant, or the characteristics of pregnant women at risk of sPTD differed. Therefore, findings on the efficacy of probiotics to prevent sPTD have been inconsistent.

*Clostridium* spp. induce the production of regulatory T (Treg) cells [18], which are essential for the maintenance of pregnancy [19-22]. Since the abundance of *Clostridium* spp. is significantly reduced in sPTD cases [14], the number of Treg cells produced may be insufficient to maintain pregnancy. Therefore, probiotics including *Clostridium* spp. have the potential to prevent sPTD in women at risk of sPTD. In 2 retrospective cohort studies, probiotics including *Clostridium* spp. reduced the recurrence of sPTD. One study showed that the rate of preterm delivery at <32 weeks of gestation was significantly reduced in pregnant women at a high risk of sPTD [23], while the other demonstrated that the rate of recurrent sPTD was significantly reduced (9.8% vs 30.1%, *P*=.002) by probiotics including *Clostridium* spp [24].

Based on this clinical and pathological background, we are planning a phase 3, prospective, single-arm trial to confirm the preventative effects of probiotics, including *Clostridium butyricum*, on sPTD in pregnant women with a history of sPTD. This trial will evaluate the rate of recurrent sPTD based on that reported in a perinatal registration database from the Japan Obstetrics and Gynecology Society for the perinatal center (22.3%) [6], which covers approximately 25% of deliveries in Japan.

## Methods

### Trial Design

**Figure 1:**
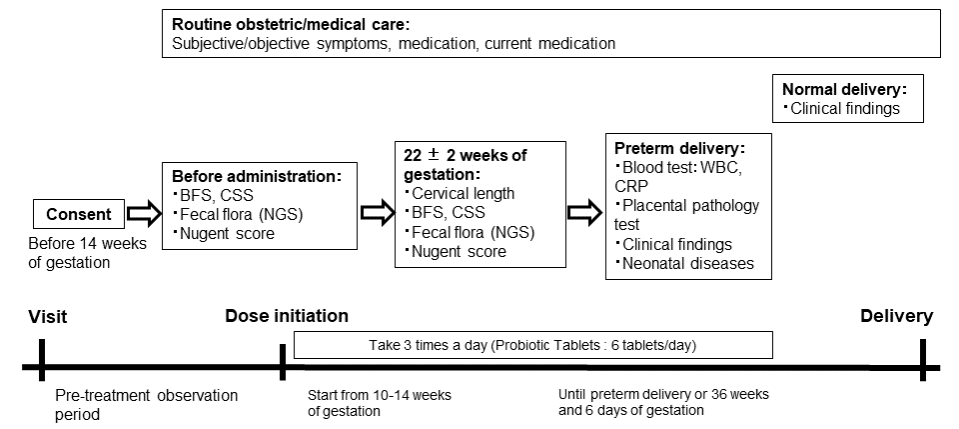
A summary of the design of the Prevention of Recurrent Spontaneous Preterm Delivery by Probiotics (PPP) trial. After informed consent, the oral administration of probiotics, including *Clostridium butyricum* (10 mg/tablet), *Enterococcus faecium* (2 mg/tablet), and *Bacillus subtilis* (10 mg/tablet), is initiated at 10-14 weeks. BFS: Bristol Stool Form Scale; CRP: C-reactive protein; CSS: constipation scoring system; NGS: next-generation sequencing; WBC: white blood cell count.

The Prevention of Recurrent Spontaneous Preterm Delivery by Probiotics (PPP) trial is designed as a prospective, single-arm, nonblinded, multicenter clinical trial to confirm the prevention of sPTD by oral probiotics in pregnant women with a history of sPTD. In Japan, 31 hospitals are participating, and the trial is scheduled to run from April 1, 2021, to March 31, 2025 (registration period was from June 1, 2021, to March 31, 2024.) The design of the trial is summarized in Figure 1. This trial will be performed according to the ethical principles originating from the Declaration of Helsinki and the Clinical Trial Act.

### Study Participants

#### Recruitment Setting

We will recruit pregnant women who meet all the inclusion criteria and do not have any of the listed exclusion criteria. In Japan, most women visit an obstetrical clinic between 5 weeks and 8 weeks of gestation. After confirming the eligibility criteria, we will register pregnant women. In this trial, a history of sPTD is defined as preterm delivery by labor pains, clinical chorioamnionitis, intrauterine infection, or histological chorioamnionitis diagnosed after preterm delivery, with or without obstetrical complications. Informed consent will be obtained from pregnant women prior to their registration. Pregnant women can review the details of this trial on the Japan Registry of Clinical Trial.

#### Inclusion Criteria

The inclusion criteria were women who had been pregnant <15 weeks, with a history of sPTD, aged 18 years to 43 years at consent, and who provided written informed consent.

#### Exclusion Criteria

The exclusion criteria included pregnant women with a history of indicated preterm birth, such as abruptio placentae, gestational diabetes mellitus, pregnancy-induced hypertension, eclampsia, HELLP (Hemolysis, Elevated Liver Enzymes, and Low Platelets) syndrome, pulmonary embolism, cerebral hemorrhage, pulmonary edema, acute fatty liver of pregnancy, placenta previa, fetal growth restriction (≤–2.0 SD), oligohydramnios, polyhydramnios, fetal diseases with chromosomal abnormalities, fetuses with multiple malformations, fetal edema, fetal dysfunction, and pregnant women with histories of sPTD for a multiple pregnancy. In addition, pregnant women with a multiple pregnancy (singleton cases after multifetal pregnancy reduction or another fetal death are also excluded), a severe physical disability, with a history of serious hypersensitivity or anaphylactic reactions caused by probiotics (including lactomin, *C. butyricum*, and amylolytic bacillus), using a medical drug or supplement including *Clostridium* spp. within 2 weeks before enrollment, with a history of cervical conization, with uterine malformation, with cervical polyps and massive genital bleeding before 10 weeks of gestation, with subchorionic hematoma and massive genital bleeding at the time of obtaining informed consent, who regularly use steroids, with diabetes mellitus, with autoimmune disease, diagnosed or treated for malignant disease at the time of informed consent (cases in which malignant disease has been completely cured may be included), with fetal morphological abnormalities at the time of informed consent, currently participating in other clinical trials or those who previously participated in a clinical trial and for whom at least 1 month has not passed since the last dose of the study drug was taken, or classified as inapplicable for this trial by the investigators are excluded.

### Study Procedures

**Figure 2:**
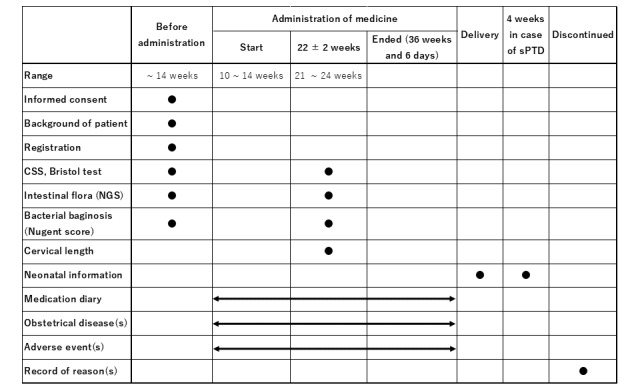
The schedule of the Prevention of Recurrent Spontaneous Preterm Delivery by Probiotics (PPP) trial. CSS: constipation scoring system; NGS: next-generation sequencing; sPTD: spontaneous preterm delivery.

The trial schedule is shown in Figure 2. All registered pregnant women will receive oral probiotics. The probiotics used are already available drugs including *C. butyricum* (10 mg/tablet), *Enterococcus faecium* (2 mg/tablet), and *Bacillus subtilis* (10 mg/tablet), because the probiotics containing *C. butyricum* alone are not commercially available in Japan. Probiotics are started from 10 weeks to 14 weeks and stopped at 36 weeks 6 days of gestation, late miscarriage, or sPTD. The maximum duration of administration is 188 days.

**Figure 3:**
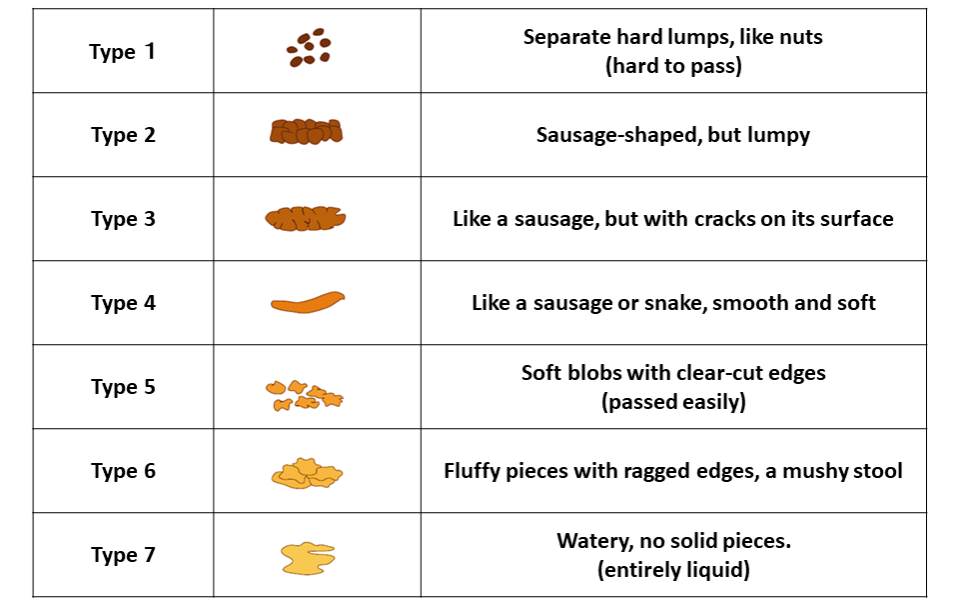
The Bristol Stool Form Scale.

Before the initiation of probiotics, stool (for the NGS) and vaginal secretion (for the Nugent score) samples will be collected, and the Bristol Stool Form Scale (BFS; Figure 3) and the constipation scoring system (CSS: Table 1) are rated. The BFS expresses the characteristics of stool as numbers: the lower the number, the less water the stool contains and the harder it is, whereas the higher the number, the more watery and soft it is. Stool is classified into 7 levels from Type 1 to Type 7, with Type 3 to Type 5 being considered the normal range of stool, and Type 4 stool being ideal. The BFS is evaluated before and during treatment. The CSS results in a subjective score of 30 points resulting from 7 items being rated on a scale from 0 to 4 and 1 item rated on a scale from 0 to 2; the items include the number of bowel movements per week, residual bowel movements, frequency of abdominal pain, and time required for bowel movements [25]. In this trial, the CSS is a modified CSS consisting of 26 points, excluding the “duration of constipation,” and is evaluated before and during treatment. At 22 ± 2 weeks of gestation, these samples will be collected again, the questionnaire will be completed for evaluations during probiotic administration, and cervical lengths will be measured.

**Table 1:** The Constipation Scoring System (total of 30 points possible).

Items evaluated	Points				
	0	1	2	3	4
Frequency of bowel movements	1-2 times per 1-2 days	2 times per week	Once per week	Less than once per week	Less than once per month
Painful evacuation effort	Never	Rarely	Sometimes	Usually	Always
Feeling of an incomplete evacuation	Never	Rarely	Sometimes	Usually	Always
Abdominal pain	Never	Rarely	Sometimes	Usually	Always
Time spent on the toilet to defecate (minutes)	<5	5-9	10-19	20-29	≥30
Type of assistance	No assistance	Stimulative laxatives	Digital assistance or enema	—^a^	—
Unsuccessful attempts to evacuate per 24 hours	0	1-3	4-6	7-9	≥10
Duration of constipation (years)	<1	1-5	6-10	11-20	≥21

^a^Not applicable.

In this trial, any treatment to prevent late miscarriage or sPTD, such as progesterone (17-alpha-hydroxyprogesterone caproate, which is used at a dosage of 125 mg and covered by the national health insurance system in Japan but is not used to prevent sPTD in the first trimester), cerclage, tocolysis, and antibiotics, could be used because there is no evidence to support their prevention of sPTD. The previously reported rate of recurrent sPTD of 22.3% in Japan [6] is presumed to be the result of these treatments.

### Endpoints

The primary endpoint is the rate of recurrent sPTD at <37 weeks of gestation. The secondary endpoints are the rate of sPTD at <34 weeks of gestation, the rate of recurrent sPTD at <28 weeks of gestation, the proportion of stillbirth, constipation (using the BFS and CSS), the ratio of intestinal *Clostridium* spp. (as measured using next-generation sequencing [NGS]), and bacterial vaginosis (using the Nugent score). In cases of sPTD, histological chorioamnionitis, or funisitis, neonatal prognosis (gestational weeks at delivery, birth weight, hospitalization in the neonatal intensive care unit [NICU], neonatal death, respiratory distress syndrome, PVL, BPD, IVH, and NEC) will be evaluated.

In this trial, to analyze the composition of the microbiota (using NGS), a stool collection brush and storage tube (Fujifilm WAKO Pure Chemicals) containing DMSO-EDTA salt solution buffer [26] was prepared, and 2 tubes were delivered to each participant. Pretreatment feces were collected in the tubes by each participant and stored under cool conditions. Posttreatment (22±2 weeks of gestation) feces were also collected. These tubes were compiled at ReLife Inc at a temperature <4°C, then transported to the Kyoto Institute of Nutrition and Pathology for analyses of the microbiota composition.

Fecal DNA was extracted using a commercial kit (QuickGene DNA tissue kit, KURABO) as described elsewhere [27]. The procedure for metagenomic 16S rRNA sequencing using Miseq (Illumina) was the same as that previously described [28]. The processing of sequencing data, including quality filtering, chimera checks, operational taxonomic unit definitions, and taxonomy assignments, was performed using QIIME1.9.1, USEARCH, and UCHIME in the same manner as previously described [28]. Alpha-diversity metrics (the Chao1 or Shannon index) were calculated using QIIME1.9.1 software. To calculate the distances between samples, beta-diversity was estimated using the UniFrac metric and visualized using a principal coordinate analysis.

### Adverse Event Reporting and Harms

As for safety evaluations, all adverse events will be reported and evaluated. Adverse events refer to any unfavorable change in clinical symptoms, laboratory data, or disease temporally associated with probiotics used in this trial, regardless of whether it is considered to be related to the trial product. However, unfavorable clinical symptoms such as constipation, which could be considered due to maternal physiological changes, are not treated as an adverse event. When an adverse event occurs, patients will receive appropriate treatment, and the cost will be supported by the national health insurance. All serious adverse events that could be assumed to be caused by probiotics used in this trial must be reported and investigated to the Clinical Research Review Board of Toyama University Hospital. In addition, the information will be shared with all investigators.

### Data Analysis

#### Sample Size Calculation

Based on the previously reported rate of recurrent sPTD at <37 weeks of gestation of 22.3% in Japan [6], we considered a clinically significant reduction in the relative risk to be 30% (risk ratio=0.7). Assuming the effect of the probiotics corresponds to a risk ratio of 0.7 (with the rates under the null hypothesis being 0.223 and under the alternative hypothesis being 0.156) and using a 1-sided test with a significance level of .025 and a power of 80%, the required sample size was calculated to be 298 participants. We considered a withdrawal rate of 10% based on the incidence of miscarriage at <22 weeks of gestation [29] and a dropout rate of 5% and, thus, set the required sample size to 345 participants.

#### Statistical Analysis

The efficacy analysis set consists of all patients enrolled in this trial except for patients who will not be treated with the protocol treatment, whose data will not be collected after the protocol treatment starts, who are designated to be ineligible after enrollment, or in whom pregnancy was terminated before 21 weeks of gestation for any reason.

The safety analysis set consists of patients who will be treated with the protocol treatment at least once. Frequencies and percentages are calculated for discrete variables, and summary statistics (mean, standard deviation, minimum, median, or maximum) are calculated for continuous variables. As the primary analysis, we will conduct a binomial test on the rate of recurrent sPTD at <37 weeks of gestation.

### Data Management

After registration, the maternal characteristics (the number of pregnant histories, the number and gestational weeks of previous miscarriage or sPTD, date of birth, age, height, weight, BMI, smoking, uterine myoma, allergy, medical history, diseases, internal medicine) will be recorded. BFS and CSS will be calculated before probiotic initiation and again at 22±2 weeks of gestation, at which time cervical length will also be recorded.

In cases of term delivery, information, such as neonatal body weight, sex, and Apgar scores, will be recorded. On the other hand, in cases of preterm delivery, more information will be recorded for the 4 weeks after delivery, including histological chorioamnionitis, funisitis, and neonatal prognosis (hospitalization in the NICU, neonatal death, respiratory distress syndrome, PVL, BPD, IVH, and NEC). All trial data are housed within a Research Electronic Data Capture (REDCap) system in Toyama University Hospital. The local principal investigator in each hospital will input the trial data online. Data management will be performed every 6 months by the data center in Toyama University Hospital.

### Monitoring

This trial is monitored to ensure the safety of pregnant women, that the protocol is being followed, and that data are being accurately collected. The monitor is not involved in this trial and is approved by the investigator.

### Ethical Considerations

The protocol for this trial was approved by the Clinical Research Review Board of Toyama University Hospital (SCR2020008) on March 31, 2021. This trial was registered in the Japan Registry of Clinical Trials on April 28, 2021. All participants will be required to sign and date an informed consent form. Each participant will be given a unique ID for the PPP trial and will be registered in the RedCap system without personal information such as their name. Participants will receive probiotic tablets at no cost but will not receive any monetary compensation.

## Results

Recruitment began on May 1, 2021. This trial is ongoing, and 345 pregnant women with a history of sPTD have been registered to date. Data collection is expected to be completed by October 2024, and the trial will finish on approximately March 31, 2025.

## Discussion

The reasons behind the effectiveness of probiotics or prebiotics for preventing sPTD have not been verified by meta-analysis [15-17]; potential reasons include differences between the characteristics of pregnant women at risk of sPTD, the types of probiotics or prebiotics, and initiation of their administration. In addition, the main probiotic used in previous research was *Lactobacillus* spp. This trial will examine the effects of probiotics, including *Clostridium* spp., which induce the production of Treg cells that play an important role in maintaining pregnancy [18-22]. There are a few reports that probiotics, including *Clostridium* spp., improved clinical symptoms in inflammatory bowel disease [30,31]. However, there is no report showing changes in intestinal flora or Treg cells. The proliferation of Treg cells in this study cannot be shown as direct evidence at present, and all theories remain a matter of speculation.

We are carrying out a prospective, single-arm, nonblinded, multicenter clinical trial to confirm the efficacy of oral probiotics including *C. butyricum*, *E. faecium*, and *B. subtilis* for the prevention of sPTD starting in the first trimester in pregnant women with a history of sPTD. *E. faecium* and *B. subtilis* can coexist symbiotically, and they increase their metabolic products and themselves [32]. In addition, *B. subtilis* and *C. butyricum* can coexist symbiotically, and they increase their metabolic products and themselves [33]. Therefore, these 3 bacteria, namely *E. faecium*, *B. subtilis,* and *C. butyricum*, might coexist symbiotically. Furthermore, proliferation of *C. butyricum* might further lead to the proliferation of *Clostridium* spp. in the human intestine [34,35]. Hence, the presence of these 3 types of bacteria is predicted to increase the proliferation of *Clostridium* spp. in the intestine.

The rate of sPTD in each country is different depending on region, ethnicity, income, and the level of medical care. Although the rate of sPTD in Japan tends to be low [1] and the recurrence of sPTD is also low [6], it is important in this single-arm trial to investigate the rate of recurrent sPTD (22.3%) using reliable data [6]. Antimicrobial agents could affect the prolongation of pregnancy. Therefore, we would like to consider excluding cases in which antibiotics were used for a long time (1 week or more). However, most cases who receive antibiotics for a long time have sPTD. Therefore, we did not exclude study participants. In addition, the use of antibiotics in the first trimester would be rare.

The first limitation of the PPP trial is that it has not been proven whether intestinal *Clostridium spp*. directly induce the proliferation of Treg cells. This trial focuses on the rate of recurrent sPTD. Second, this is a single-arm trial with no placebo group. This clinical trial involves pregnant women and requires the administration of oral medicine for a long time, approximately 6 months. Although a randomized controlled trial (RCT) is considered the gold standard for evaluating the efficacy of probiotics, pregnant women and children are considered a high-risk group for RCTs. In such trials, real-world data could be used and analyzed for comparison [36]. If the results of this single-arm trial show a significant reduction in the rate of recurrent sPTD, an RCT may be performed in the near feature. Probiotics are not expensive, and their use is already widespread. Therefore, the use of probiotics to prevent recurrent sPTD may be easily used in many countries. In lower-income countries with a high rate of sPTD, probiotics could contribute to the prevention of recurrent sPTD.

## Abbreviations

BFS: Bristol Stool Form Scale
BPD: bronchopulmonary dysplasia
CSS: constipation scoring system
HELLP: Hemolysis, Elevated Liver Enzymes, and Low Platelets
IVH: intraventricular hemorrhage
NEC: necrotizing enterocolitis
NGS: next-generation sequencing
NICU: neonatal intensive care unit
PPP: Prevention of Recurrent Spontaneous Preterm Delivery by Probiotics
PVL: periventricular leukomalacia
RCT: randomized controlled trial
RDS: respiratory distress syndrome
REDCap: Research Electronic Data Capture
sPTD: spontaneous preterm delivery
Treg: regulatory T

## Data Availability

The data set used and analyzed in this study will be available from the corresponding author upon reasonable request.
